# Identification of novel biomarkers in breast cancer via integrated bioinformatics analysis and experimental validation

**DOI:** 10.1080/21655979.2021.2005747

**Published:** 2021-12-13

**Authors:** Ningning Wang, Haichen Zhang, Dan Li, Chunteng Jiang, Haidong Zhao, Yun Teng

**Affiliations:** a Department of Food Nutrition and Safety, School of Public Health, Dalian Medical University, Dalian, P.R. China; b Department of Radiation Oncology, The Second Hospital of Dalian Medical University, Dalian, P.R. China; c Department of Breast Surgery, The Second Hospital of Dalian Medical University, Dalian, P.R. China; d Department of Internal Medicine, The Affiliated Zhongshan Hospital of Dalian University, Dalian, P.R. China; e Department of Cardiology and Pneumology, University Medical Center of Göttingen, Georg-August University of Göttingen, Lower Saxony, Germany

**Keywords:** Breast cancer, differentially expressed genes, survival, bioinformatics analysis, experimental validation

## Abstract

Breast cancer (BC), an extremely aggressive malignant tumor, causes a large number of deaths worldwide. In this study, we pooled profile datasets from three cohorts to illuminate the underlying key genes and pathways of BC. Expression profiles GSE42568, GSE45827, and GSE124646, including 244 BC tissues and 28 normal breast tissues, were integrated and analyzed. Differentially expressed genes (DEGs) were screened out based on these three datasets. Functional analysis including Gene Ontology (GO) and Kyoto Encyclopedia of Gene and Genome (KEGG) pathway were performed using The Database for Annotation, Visualization and Integrated Discovery (DAVID). Moreover, Cytoscape with Search Tool for the Retrieval of Interacting Genes (STRING) and Molecular Complex Detection (MCODE) plugin were utilized to visualize protein protein interaction (PPI) of these DEGs. The module with the highest connectivity of gene interactions was selected for further analysis. All of these hub genes had a significantly worse prognosis in BC by survival analysis. Additionally, four genes (CDK1, CDC20, AURKA, and MCM4) dramatically were enriched in oocyte meiosis and cell cycle pathways through re-analysis of DAVID. Moreover, the mRNA and protein levels of CDK1, CDC20, AURKA, and MCM4 were significantly increased in BC patients. In addition, knockdown of CDK1 and CDC20 by small interfering RNA remarkably suppressed cell migration and invasion in MCF-7 and MDA-MB-231 cells. In conclusion, our results suggested that CDK1, CDC20, AURKA, and MCM4 were reliable biomarkers of BC via bioinformatics analysis and experimental validation and may act as prospective targets for BC diagnosis and treatment.

## 1. Introduction

Breast cancer (BC) is the most widely recognized malignancy that forces enormous well-being troubles among women worldwide [1]. In terms of its annual incidence (currently 17 million cases), it is expanding alarmingly [2]. In 2019, around 268,600 new cases of BC were reported in the United States, resulting in 41,760 deaths [3]. Despite new advancements in therapeutic strategies BC in recent years, the treatment of BC has become more effective, and the mortality rate of BC has been significantly reduced. However, the recurrence, metastasis, and rapid dissemination of BC have not been completely controlled, and have become a huge obstacle in clinical therapy. Hence, it is extremely necessary to seek more credible prognostic biomarkers as targets for better understanding the potential mechanisms, improve the treatment effect and reduce distant metastasis, thereby promoting survival rate.

To date, a number of biomarkers have been implemented for screening, diagnosing, and monitoring the recurrence of BC. For instance, human epidermal growth factor receptor 2 (HER2) is overexpressed in 15% of BC patients and was identified as a biomarker of poor prognosis a quarter century ago [4]. The antigen KI-67, as encoded by the MKI67 gene, is a critical cell proliferation-related biomarker and has been used clinically as a prognostic indicator of tumor recurrence and clinical outcome [5,6]. Moreover, miRNAs that perform as co-transcriptional regulators are likely to have positive and negative associations with their target mRNAs in BC. It has been discovered that BC patients with a higher expression of miR-1307-3p, miR-940, and miR-340-3p had a worse overall survival [7]. The expression alteration of hsa-miR-503, hsa-miR-1307, hsa-miR-212, and hsa-miR-592 are strongly associated with the prognosis of BC [8]. However, the estimation of KI-67 and these miRNAs in BC has not yet been extensively applied as biomarkers in the clinic due to the lack of reproducibility. Hence, it is essential to seek more specific biomarkers to improve the accuracy of BC diagnosis.

Current developments in microarray and sequencing technologies can now simultaneously screen hundreds of genes that are dysregulated at the transcriptional level in tumors and play a vital role in oncogenesis and development [9]. Multiple underlying prognostic biomarkers and pharmaceutical targets can be discovered through the joint analysis of gene expression profiles and clinical data. In order to identify reliable biomarkers of BC, three transcriptome microarray datasets of BC-related differentially expressed genes (DEGs) from Gene Expression Omnibus (GEO) database were analyzed. The molecular mechanisms underlying the development of BC were addressed by Gene Ontology (GO) function, Kyoto Encyclopedia of Gene and Genome (KEGG) pathways, and protein–protein interaction (PPI) network analysis. The Kaplan Meier plotter database was utilized to analyze overall survival in BC. Following this, a re-analysis of hub genes was performed and four genes were involved with important biological processes and pathways in BC. Meanwhile, we further performed an analysis of the expression of these four genes in the patients’ breast cancer tissues. Functional experiments were applied to validate their capacity to impact migration and metastasis in BC development.

In this study, we obtained transcriptome data from GEO database and constructed a mRNA-based signature to explore its role in prediction of BC prognosis. We hypothesized that carcinogenicity of hub genes correlated with poor prognosis of BC. Our data aimed to unveil potential prognostic indicators and therapeutic targets for BC. This may provide insight into the mechanisms underlying the onset, development, and deterioration of BC, and bring new targets for clinical diagnosis and treatment of BC.

## 2. Material and methods

### 2.1. Data source

Gene expression profiles of GSE42568, GSE45827, and GSE124646 were obtained from GEO database. GSE42568 and GSE45827 were based on the platform GPL570 ([HG-U133_Plus_2] Affymetrix Human Genome U133 Plus 2.0 Array), and GSE124646 was based on the platform GPL96 ([HG-U133A] Affymetrix Human Genome U133A Array), which included 104 BC tissues and 17 normal breast tissues, 130 BC tissues and 11 normal breast tissues, and 10 BC tissues and 10 normal breast tissues, respectively.

### 2.2. Identification of DEGs

DEGs were screened out of BC and normal breast tissue samples by using the GEO2R online tool. DEGs with |log FC| > 2 and adjust *P* value < 0.05 were considered as standard criterion [10]. Each profile of DEGs was downloaded and overlapped using Venn diagram software. The DEGs with log FC < 0 was regarded as down-regulated genes, and vice versa.

### 2.3. Functional enrichment analysis

GO is a typical approach for distinguishing characteristic biological attributes for high-throughput transcriptome data [11]. KEGG is a data repository for handling genomes, biological pathways, diseases, medications, and chemical substances [12]. The Database for Annotation Visualization and Integrated Discovery (DAVID) is an online bioinformatic website for differentiating the function of numerous or proteins, and integrating this information to decipher the GO and KEGG pathway of identified DEGs [13].

### 2.4. PPI network and module establishment

Search Tool for the Retrieval of Interacting Genes (STRING) is an online method assessing the information of PPI [14]. Cytoscape is an bioinformatics software, which was applied to exam and trace the potential correlation between these DEGs, and visualize molecular interaction networks [15]. Functional module analysis was conducted using MCODE plugin to cluster a given network to a densely connected territory based on topology. The selection criterion was established as follows: MCODE scores >5, degree cutoff = 2, node score cutoff = 0.2, max depth = 100 and k-score = 2 [16].

### 2.5. Survival analysis

Kaplan–Meier plotter is a website vehicle that can predict the impact of genes on survival. By entering the genes of interest to the blank of the website, patients were sorted into two groups on the basis of hub gene expression levels, and statistically analyzed the survival rate. The log rank *P* value and hazard ratio (HR) with 95% confidence intervals were calculated and displayed [17]. The Kaplan Meier plotter database was utilized to pinpoint hub genes with high connectivity to analyze overall survival in BC.

### 2.6. Interrelation analysis

The expression of four hub genes mRNA in BC and interrelation between genes were analyzed using the Breast Cancer Gene-Expression Miner v4.7 (bc-GenExMiner) [18]. The interrelation between these four genes were generated using the correlation module.

### 2.7. Further authentication of hub genes using other open databases

To verify the significant values of four hub genes, mRNA-seq data for BC was downloaded from The Cancer Genome Atlas (TCGA) database. The expression of these four genes based on healthy, BC-adjacent and BC samples were visualized using the Breast Cancer Gene-Expression Miner v4.7 [19].

### 2.8. Cell culture

Human breast carcinoma lines (MCF-7, and MDA-MB-231), and normal human mammary epithelial cell line (MCF-10A) were purchased from American Type Culture Collection center (ATCC, Manassas, VA, USA). MCF-7 and MDA-MB-231 cells were grown in Dulbecco’s modified Eagle’s medium (DMEM, Hyclone, Logan, UT, USA), supplemented with 10% fetal bovine serum (FBS, Hyclone, Logan, UT, USA), 100 U/mL penicillin and 100 mg/mL streptomycin (Sigma Aldrich, St. Louis, MO, USA), and MCF-10A were cultured in DMEM/Ham’s F-12 (Invitrogen, Carlsbad, CA) supplemented with 100 ng/ml cholera toxin, 20 ng/ml epidermal growth factor (EGF), 0.01 mg/ml insulin, 500 ng/ml hydrocortisone, and 5% chelex-treated horse serum. These cells are incubated in a humidified atmosphere 5% CO_2_ at 37°C incubator (Thermo Fisher Scientific, Waltham, MA, USA).

### 2.9. Transfection assays

MCF-7 and MDA-MB-231 cells were seeded into 6-well plates. The cells reached desired density and were transfected with their negative controls or siRNAs against CDK1 and CDC20 (Gene Pharma, Shanghai, China) by lipofectamine 3000 reagent (Thermo Fisher Scientific, Waltham, MA, USA). After transfection overnight, cells were cultured for additional 48 h in fresh medium and harvested for various assays.

### 2.10. Human tissue samples

The study included 10 female patients and 10 age- and gender-matched controls. Samples from patients with BC and normal breast tissues were obtained from The Second Hospital of Dalian Medical University and confirmed by pathological examination. The study was performed in accordance with the Declaration of Helsinki and approved by the Ethics Committee of The Second Hospital of Dalian Medical University. Patients provided written consent.

### 2.11. Western blot analysis

The total proteins were extracted and completely lysed from the frozen breast cancer tissues or cell lines by RIPA reagent (Invitrogen, Carlsbad, CA, USA) with inhibitors. Bicinchoninic acid (BCA) method was applied to quantify with the protein concentration. 30 µg of proteins were loaded into each well and separated on 15% sodium dodecyl sulfate polyacrylamide gel electrophoresis (SDS-PAGE) gels, and then transferred onto polyvinylidene difluoride (PVDF) membranes. Membranes were blocked with 5% nonfat milk in TBST buffer for 1 h at 37°C. Then, the membranes were incubated with the following primary antibodies against CDC20 (Abcam, Cambridge, UK), CDK1 (Abcam, Cambridge, UK), AURKA (Cell signaling Technology, Danvers, MA, USA), MCM4 (Abcam, Cambridge, UK) and the internal control GAPDH (ZSGB-BIO, Beijing, China) overnight at 4°C. After being washed three times with TBST, the membranes were incubated with horse-radish peroxidase (HRP)-conjugated as secondary antibodies for 1 h at room temperature, and the protein bands were visualized by chemiluminescence reagent (Thermo Scientific, Waltham, MA, USA) and imaging system (Bio-Rad Laboratory, Hercules, CA, USA). Relative protein expression was measured by Image J software.

### 2.12. RNA isolation, cDNA synthesis and quantitative real-time polymerase chain reaction (RT-PCR)

RNA was extracted from breast tissues using RNAiso Plus Kit (Takara, Kyoto, Japan), then cDNA synthesis was executed by using the PrimeScript TM RT reagent Kit (AG11705, Accurate Biotechnology, Hunan, China). RT-PCR was performed using a Rotor-Gene Q instrument (Qiagen, Dusseldorf, Germany) with SYBR Premix Ex TaqTM (AG11701, Accurate Biotechnology, Hunan, China). The relative gene expression was calculated by the ΔΔCt method. The sequences of RT-PCR primers were used as follows: CDK1: forward 5ʹ-CCTTTAGCGCGGATCTACC-3ʹ and reverse 5ʹ-GGAACCCCTTCCTCTTCACT-3ʹ; CDC20: forward 5ʹ-AAAATGCGCCAGAGGGTTAT-3ʹ and reverse 5ʹ-GCTTGCACTCCACAGGTACA-3ʹ; MCM4: forward 5ʹ-ATGGCGGTGCTAAAGGACTA-3ʹ and reverse 5ʹ-CTGTCGAGGGTATGCAGAAA-3ʹ; AURKA: forward 5ʹ-GGGTCTTGTGTCCTTCAAATTC-3ʹ and reverse 5ʹ-TGCTTGCTCTTTTGGGTGTTA-3ʹ; GAPDH: forward 5ʹ-GTCTCCTCTGACTTCAACAGCG-3ʹ and reverse 5ʹ- ACCACCCTGTTGCTGTAGCCAA-3ʹ.

### 2.13. Immunohistochemistry (IHC) analysis

5 μm thickness of paraffin-embedded breast tissue sections were deparaffinized in xylene and hydrated in graded 100%, 95%, 85%, 75%, 60%, and 30% ethanol. Afterward, sections were treated by antigen retrieval in sodium citrate buffer (10 mM citric acid, pH 6.0) in an oven for 1 h at 60C. Then slides were cooled down to room temperature and washed with PBS, and was quenched by incubation in 3% H_2_O_2_ for 10 min. After that, the slides were washed in PBS and incubated in goat serum for 1 h. Then the slides were incubated with primary antibodies CDC20 (1:100 dilution) and CDK1 (1:100 dilution) overnight at 4°C. Slides were then washed in PBS and incubated with the secondary antibody (1:300 dilution) for 1 h at room temperature. After that, slides were washed in PBS and incubated for 30 min in the IHC kit (SP-9000-D, ZSGB-BIO, Beijing, China). After washed in PBS, the slides were incubated in diaminobenzidine (DAB) followed by counterstaining with hematoxylin. Images of antigen distribution were captured under microscope. Representative immunohistochemical staining of CDC20 and CDK1 in the breast tissues were from 3 human normal tissues and 3 BC tissues.

### 2.14. Cell migration and invasion assays

For the transwell migration assay, MCF-7 and MDA-MB-231 cells were transfected with negative controls or siRNA against CDK1 or CDC20. After 48 hours of transfection, cells were harvested and resuspended with 100 µl of serum-free medium, then the same number of cells were plated into the upper chamber (CORNING, New York, USA) with a non-coated membrane. For the invasion assay, the same number of cells in serum-free medium were plated into the upper chamber with a Matrigel (BD Biosciences, Franklin Lakes, NJ, USA). 600 µl medium containing 10% FBS was filled into the lower chambers. After overnight incubation, non- invasive cells were manually debrided from the upper surface of the upper chamber with a cotton swab; the cells on the lower surface of filters were fixed in methanol for 30 min and stained with 0.1% crystal violet for 30 min. Quantities of invaded cells was enumerated under microscope (5 fields per chamber) [20].

### 2.15. Statistical analysis

All experiments were repeated at least three times. The data were presented as means ± standard deviation (SD) and analyzed by SPSS software. One-way ANOVA followed by Student-Newman Keuls (SNK) test was used to compare differences, and *P* value < 0.05 was considered statistically significant.

## 3. Results

### 3.1. Identification of DEGs in BC

**Figure 1.**
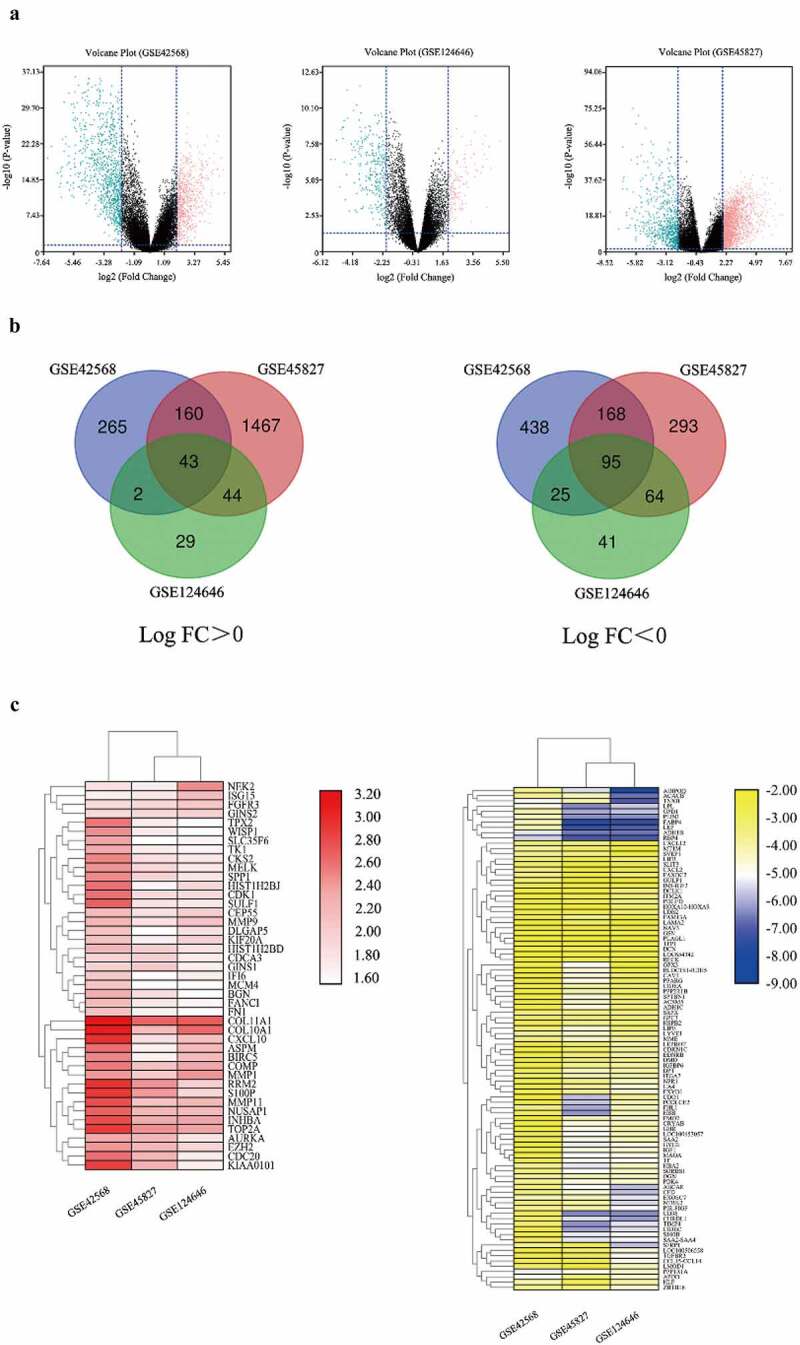
Bioinformatic analysis of the DEGs obtained from GSE42568, GSE45827, and GSE124646 datasets in BC tissues compared to the normal breast tissues. Fold change > 2 and adjust *P*-value < 0.05 as selection criteria for DEGs. (a) Volcano plot identified the DEGs in three datasets. Red dots stand for up-regulated genes and turquoise dots stand for down-regulated genes. (b) Venn diagram showed the common up-regulated and down-regulated DEGs in three datasets. (c) Heatmap showed the common DEGs in three datasets. Left heatmap indicated the common up-regulated DEGs, and right heatmap indicated the common down-regulated DEGs

After thoroughly searching in the GEO database according to the eligibility criteria, three genome-wide gene expression datasets involving BC and normal breast tissues were finally obtained. We picked up 1196 DEGs, 2334 DEGs and 343 DEGs from GSE42856, GSE45827, and GSE124646 datasets via the GEO2R online tools, respectively. In addition, we visualized the expression of DEGs in the three datasets by using volcano plots (Figure 1(a)). Gene comparison analysis was performed on these three groups, the overlap among the three datasets included 138 genes as displayed in the Venn diagram and heatmap (Figure 1(b,c), Table 1), comprising 43 up-regulated genes and 95 down-regulated genes between BC tissues and normal breast tissues.

**Table 1.** The different expression genes (DEGs) in BC. DEGs with log FC > 2 and adjust *P* value < 0.05 were considered as up-regulated genes, and DEGs with log FC < −2 and adjust *P* value < 0.05 were considered as down-regulated genes

DEGs	Genes Name
Up-regulated	TPX2, S100P, GINS1, BIRC5, EZH2, CDK1, FGFR3, AURKA, FN1, SPP1, MELK, CDC20, HIST1H2BJ///HIST1H2BG, BGN, MMP1, CKS2, ISG15, MMP11, TK1, ASPM, INHBA, CDCA3, IFI6, CEP55, RRM2, SLC35F6///CENPA, TOP2A, COMP, FANCI, MCM4, WISP1, DLGAP5, CXCL10, SULF1, KIF20A, HIST1H2BD, COL10A1, COL11A1, KIAA0101, NEK2, GINS2, NUSAP1, MMP9
Down-regulated	IGF1, CHRDL1, LAMA2, LEP, HOXA9, CES1, GHR, MAOA, CRYAB, CD36, GPD1, TF, DCLK1, NPR1, SPTBN1, LIPE, SAA1, PPARG, FIGF, PPP2R1B, MT1M, HLF, FHL1, PLAGL1, ABCA8, SVEP1, HSPB2, GSN, PDGFD, LMOD1, ZBTB16, CCL14, EDNRB, SLIT3, CIDEA, ADH1C, CIDEC, S100B, MME, MATN2, GYG2, PDK4, FAM13A, SAA4, ACSM5, PLIN1, HBA2///HBA1, DPT, OGN, IGF2, RBP4, NTRK2, CDO1, CA4, EXOSC7///CLEC3B, SORBS1, CXCL2, LIFR, LOC654342///LOC645166, LYVE1, IGFBP6, TNXB///TNXA, LEPR, CXCL12, APOD, LDB2, RECK, CAV1, RDH5, HBB, ITM2A, SRPX, TFPI, CDKN1C, FMO2, NAV3, TGFBR3, ADH1B, LPL, FABP4, FAXDC2, GPC3, ACACB, DMD, PPP1R1A, GPX3, FXYD1, ITGA7, DCN, TIMP4, PCOLCE2, SFRP1, GULP1, CFD, ADIPOQ

### 3.2. GO enrichment and KEGG analyses of DEGs

**Figure 2.**
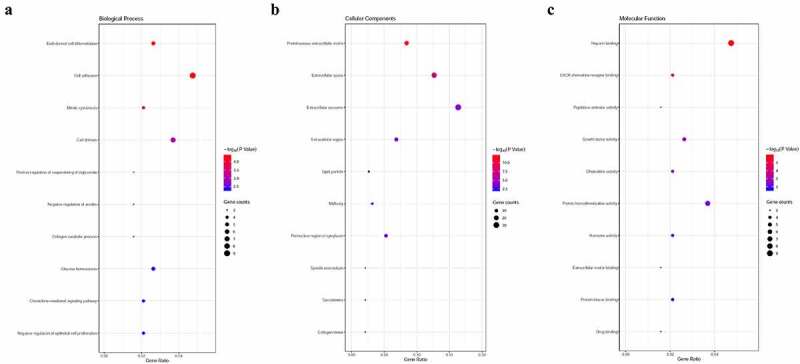
GO enrichment analysis of common DEGs associated with BC. (a) Cellular component. (b) Biological process. (c) Molecular function

To explore the systematic characters and biological functions of the identified DEGs, GO enrichment and KEGG pathway were adopted. It was indicated that the DEGs were primarily enriched in the undermentioned subcategories: endodermal cell differentiation, cell adhesion and cell division in the biological process (BP), proteinaceous extracellular matrix, extracellular space and extracellular exosome in the cellular components (CC), and heparin binding, protein homodimerization activity and growth factor activity in the molecular function (MF) by GO analysis (Figure 2(a-c)). Moreover, it was unveiled that the DEGs were enriched in signal transduction by KEGG pathway analysis (*P* < 0.05, Table 2).

**Table 2.** Kyoto Encyclopedia of Genes and Genomes (KEGG) pathway analysis in BC

Pathway	Count	Genes
PPAR signaling pathway	8	FABP4, MMP1, ADIPOQ, LPL, PPARG, PLIN1, SORBS1, CD36
AMPK signaling pathway	9	LIPE, PPP2R1B, LEP, ADIPOQ, LEPR, PPARG, CD36, IGF1, ACACB
ECM-receptor interaction	7	COMP, LAMA2, COL11A1, SPP1, FN1, ITGA7, CD36
Focal adhesion	9	COMP, LAMA2, COL11A1, PDGFD, CAV1, SPP1, FN1, ITGA7, IGF1
PI3K-Akt signaling pathway	11	GHR, COMP, LAMA2, PPP2R1B, COL11A1, PDGFD, SPP1, FN1, ITGA7, IGF1, FGFR3
Cytokine-cytokine receptor interaction	9	GHR, CCL14, CXCL10, CXCL12, LEP, LEPR, LIFR, INHBA, CXCL2
Adipocytokine signaling pathway	5	LEP, ADIPOQ, LEPR, CD36, ACACB
Pathways in cancer	11	CXCL12, EDNRB, LAMA2, MMP1, ZBTB16, CKS2, FN1, BIRC5, PPARG, IGF1, FGFR3
Regulation of lipolysis in adipocytes	4	LIPE, FABP4, NPR1, PLIN1
Proteoglycans in cancer	7	CAV1, HSPB2, IGF2, FN1, GPC3, IGF1, DCN
Oocyte meiosis	5	CDC20, PPP2R1B, CDK1, IGF1, AURKA
Drug metabolism – cytochrome P450	4	ADH1C, MAOA, ADH1B, FMO2

### 3.3. PPI network and modular selection

**Figure 3.**
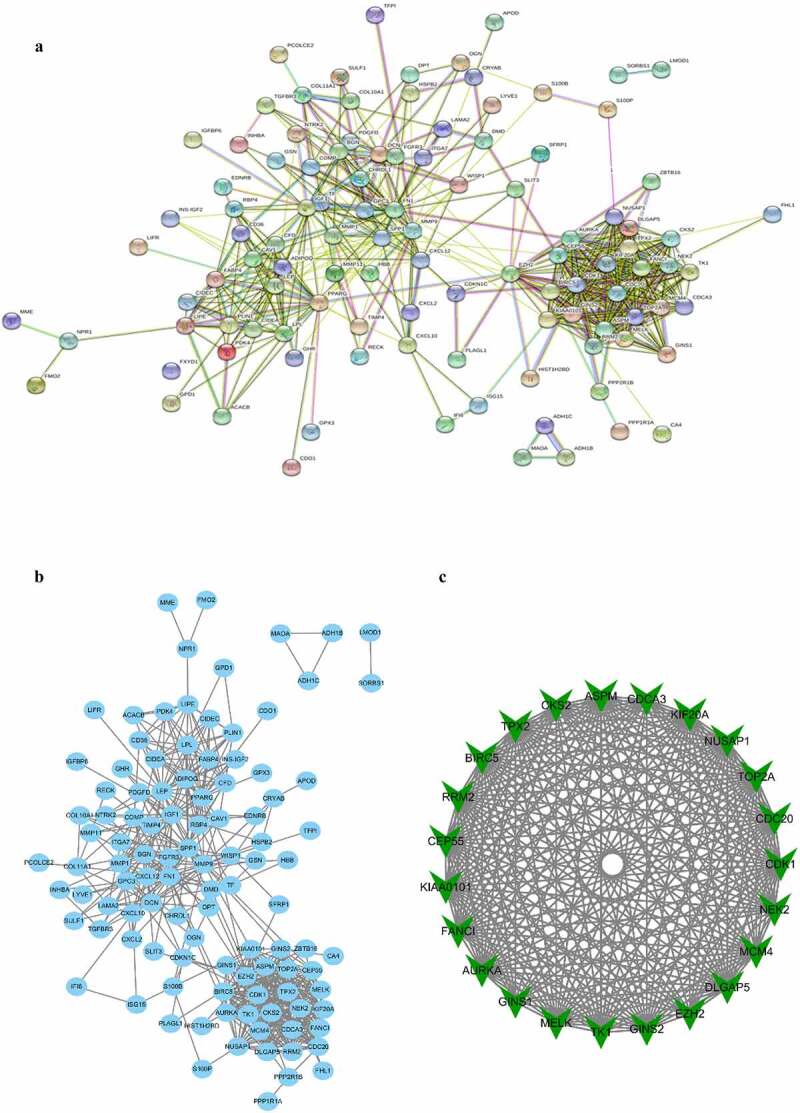
Protein–protein interaction (PPI) network complex and modular analysis of DEGs. (a) The PPI network was structured by STRING online database. (b) Total of 138 DEGs were uploaded into the PPI network complex. (c) The most highly connectivity module was picked up

In all, there were 138 DEGs uploaded into the STRING, and the PPI network was constructed (Figure 3(a-b)). In addition, MCODE, a plugin of Cytoscape software, was applied for analyzing the whole PPI network. There are five modules constructed in total. We screened out and selected the module with the highest connectivity for further analysis, containing 23 hub genes (Figure 3(c)). These hub genes were found to have the highest connectivity in the PPI network, indicating these hub genes are the most densely connected to BC.

### 3.4. Survival analysis

**Figure 4.**
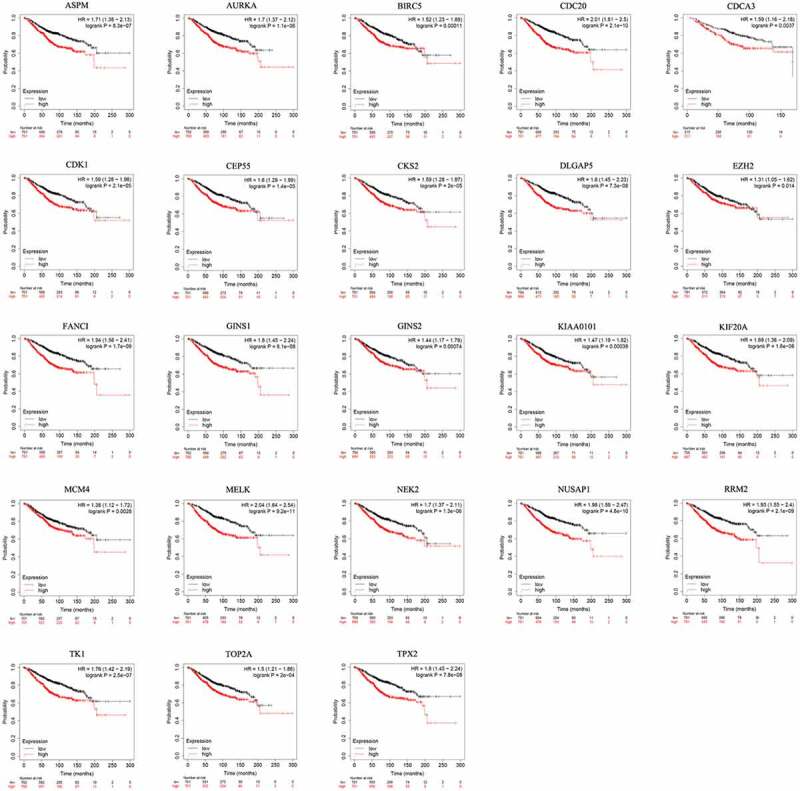
Prognostic value of the 23 hub genes in BC patients based on Kaplan-Meier Plotter. The patients were split into high and low expression groups based on the median gene expression. 23 hubs genes were analyzed for their prognostic value

To assess the prognostic value of 23 hub genes selected by MCODE in patients with BC by evaluation of the correlation between gene expression and overall survival, Kaplan–Meier plotter was applied. Intriguingly, all of hub genes with high expression had a worse overall survival in BC patients (*P* < 0.05, Figure 4). These data implies that high expression of these hub genes has a negative correlation with overall survival in BC.

### 3.5. KEGG pathway enrichment re-analysis for 23 hub genes

**Figure 5.**
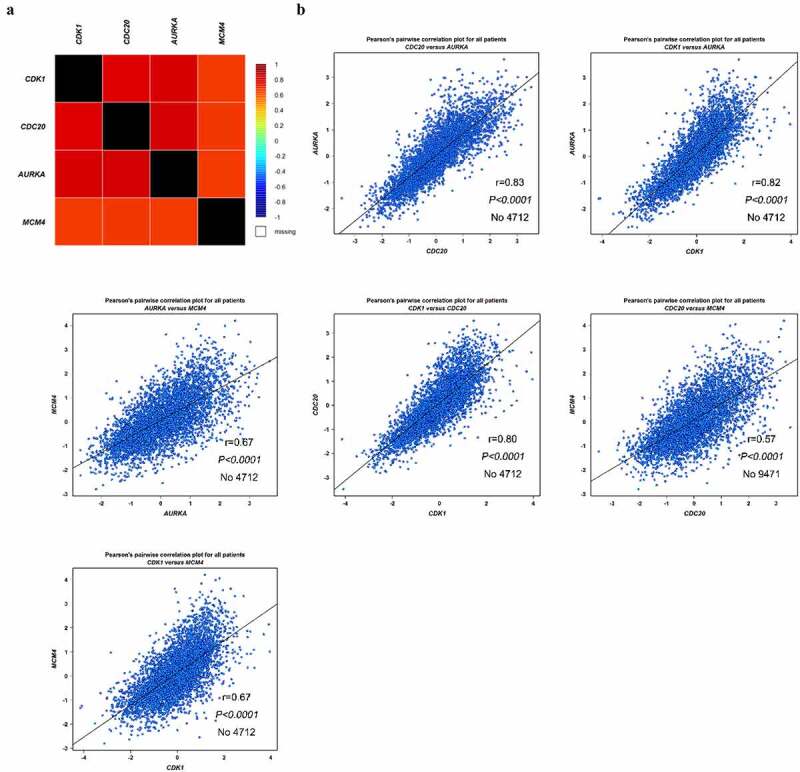
Co-expression analysis of CDK1, CDC20, AURKA and MCM4. (a-b) Analysis of the interrelation expression of these four genes in BC was performed by bc-GenExMiner software

**Figure 6.**
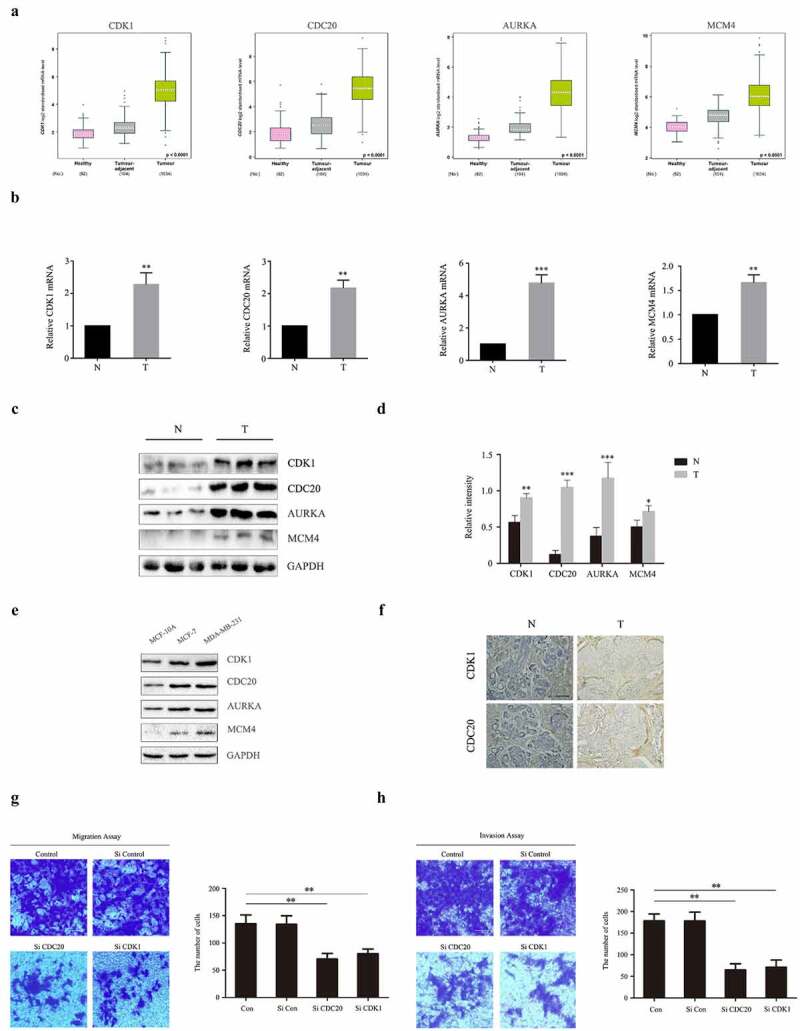
Experimental validation of CDK1, CDC20, AURKA and MCM4 expression both in human BC patients, MCF-10A, MCF-7 and MDA-MB-231 cells. (a) Analysis of mRNA levels of CDK1, CDC20, AURKA and MCM4 in healthy, BC-adjacent and BC tissues in TCGA database. (b) mRNA levels of CDK1, CDC20, AURKA and MCM4 in human samples, performed by RT-PCR. N: normal breast tissues; T: tumor tissues. (c-d) The expression of CDK1, CDC20, AURKA and MCM4 proteins in human samples. The protein fraction was analyzed by Western blot. Relative expression of these proteins was normalized by GAPDH. (e) The expression of CDK1, CDC20, AURKA and MCM4 proteins in MCF-10A, MCF-7 and MDA-MB-231 cells. (f) CDK1 and CDC20 immunohistochemistry staining of breast sections in human samples. (Scale bar = 10 μm). (g-h) MCF-7 were transfected with siRNA against human CDK1 (siCDK1) and CDC20 (siCDC20) or scrambled control siRNA, then incubated for 48 hours. Transwell and invasion assay were used for observing migration and invasion abilities of these cells. The images displayed the migrated and invaded cells into the lower chamber. (Scale bar = 10 μm). Quantified by counting the number of migrated and invaded cells in five randomly fields. *P < 0.05, **P < 0.005, ***P < 0.001, compared with the control group

To explore the possible signaling pathways involved in these 23 hub genes, the KEGG pathway was re-analyzed by DAVID (*P* < 0.05). As shown in Table 3, four genes (CDK1, CDC20, AURKA and MCM4) strikingly enriched in oocyte meiosis and cell cycle (*P* <0.05), demonstrating that these four genes are closely tied to BC. We also analyzed the expression levels of these four genes in TCGA database, which were significantly increased in BC-adjacent and BC patients compared to healthy individuals (Figure 6(a)). To further study the underlying mechanism of these four genes in BC, we performed co-expression data mining of these four genes by bc-GenExMiner software. As shown in Figure 5(a), all four genes were up-regulated in BC tissues. Besides, there was a strong positive correlation between these four genes (all *P* < 0.01, Figure 5(b)).

**Table 3.** Re-analysis of 23 selected genes via KEGG pathway enrichment

Term	Description	Genes
cfa04114	Oocyte meiosis	CDK1, CDC20, AURKA
cfa04110	Cell cycle	CDK1, CDC20, MCM4
cfa04115	p53 signaling pathway	CDK1, RRM2

### 3.6. The hub genes were verified within BC tissues

According to the bioinformatics analysis results, CDK1, CDC20, AURKA and MCM4 are four biomarkers associated with the prognosis of BC. To pursue further studies on the expression levels of these four genes, we randomly selected three each from 10 normal breast tissues and 10 BC tissues of human patients for analysis. As represented in Figure 6(b-d), in BC tissues, both mRNA and protein levels of CDK1, CDC20, AURKA and MCM4 were significantly increased compared to the normal breast tissues. Moreover, protein levels of CDK1, CDC20, AURKA and MCM4 in MCF-7 and MDA-MB-231 BC cell line were higher than those in MCF-10A normal human mammary epithelial cell line (Figure 6(e)). Additionally, IHC showed that CDK1 and CDC20 were mainly up-regulated in the cytoplasm in BC tissues (figure 6(f)). These outcomes are consistent with the sequencing results, implying these four genes act as oncogenes that play carcinogenesis roles in BC progression.

### 3.7. Knockdown of CDK1 and CDC20 decrease the capacities of migration and invasion in MCF-7 and MDA-MB-231 cells

Among the four hub genes, previous studies have confirmed that CDK1 and CDC20 were two genes involved in tumors proliferation and metastasis [21,22]. Therefore, we hypothesized CDK1 and CDC20 probably have the potential to mediate BC metastasis. To evaluate the effect of CDK1 and CDC20 on cell migration and invasion, the transwell assay was performed. Here, we adopted MCF-7 and MDA-MB-231 cell line to check the role of CDK1 and CDC20 in BC metastasis. As demonstrated in Figure 6(g-h) and supplementary 1, upon transfection of cells with CDK1-siRNA or CDC-siRNA, the capacities of migration and invasion were significantly decreased compared to untreated group. These data show that CDK1 and CDC20 play a role as tumor activators in BC metastasis.

## 4. Discussion

Breast cancer (BC), a highly heterogeneous carcinoma, is a serious menace to the health of women and is the leading cause of death in women of 40–55 years old. Despite improvements in clinical diagnosis and therapy, the rates of BC recurrence and metastasis remain extremely high due to its highly malignant character. The identification of novel biomarkers for BC is crucial to its diagnosis, therapy, and prognosis.

In the last decade, the rapid development of high-throughput techniques and public gene databases enables to filter out a wider range of disease-associated genes on the basis of abundant data by utilization of microarrays, analyze them holistically, and thus identify potential new drug targets for early diagnosis and treatment. Through analysis of the public database, seven hub genes (UQCR11, UBE2N, ADD1, TLN1, IRAK3, LY96, and MAP3K1) have recently been found to be strongly associated with familial hypercholesterolemia and contribute to a higher risk of atherosclerosis [23]. By comparing the functional annotations of four crucial genes, CCL5, ALK, TAC1, CD74, and HLA-DOA, Udhaya Kumar et al. these genes could be significantly influential in the molecular pathogenesis of emphysema [24]. Wan and associates have suggested that lncRNA ADAMTS9-AS1 not only serves as a biomarker, but also as a potential therapeutic target in prostate cancer by bioinformatics analysis and experimental validation [25]. Moreover, a study reported that identification of potential immunogenomic signatures could predict the prognosis of patients with lung squamous cell carcinoma through computational biology [26]. The use of bioinformatics methods to screen potential hub genes and improve prognosis by early diagnosis and intervention is of great interest to clinicians. In addition, previous studies also believed that using bioinformatics methods to seek novel biomarkers for BC can improve diagnostic accuracy and prognosis. According to an analysis of breast cancer public databases, COL10A1 and PITX1 have been considered as predictive biomarkers for the prognosis of BC [27,28]. In BC tissues, tumor suppressor ARHGEF10 and the oncogene SRFS1 were regarded to be negatively and positively co-regulated by miR-106b-5p, miR-106a-5p, miR-671-5p, and miR-590-3p [7]. Therefore, seeking novel biomarkers of BC remains an urgent matter.

In this study, three datasets GSE42568, GSE45827 and GSE124646 were obtained from the GEO database. In total, there were 138 BC-related genes co-expressed in these datasets, consisting of 43 up-regulated genes and 95 down-regulated genes. To gain more insight into the function of these genes, overlapping DEGs were mainly associated with cell adhesion in BP, extracellular exosome in CC and heparin binding in MF via GO analysis. KEGG pathway enrichment showed that these DEGs were primarily enriched in signal transduction. These discoveries are in high concordance with previous documented studies, suggesting the key roles of cell adhesion, extracellular exosome and heparin binding in the progression of BC [29–31]. Besides, analysis of these enriched pathways may furnish potential strategies for the development of new therapeutic agents. Afterward, PPI analysis was performed to ferret out the network of PPI, which revealed a global atlas of these crucial genes. Of which, 23 genes with higher connectivity were regarded to be the hub genes. Notably, high expression of these 23 genes resulted in a significantly poorer survival rate in BC via Kaplan Meier plotter analysis. Upon re-analyzation of 23 genes in KEGG pathway enrichment via DAVID, we found that four genes (CDK1, CDC20, AURKA and MCM4) enriched in oocyte meiosis and cell cycle.

CDK1, also known as cell division control protein 2 (CDC2), is a member of the cyclin-dependent kinase family driving the main occurrences of the cell cycle in eukaryotic cells [32]. As shown in our results, CDK1 is also mainly involved in the cell cycle. A wealth of evidence has documented that CDK1 is up-regulated as a host modulator of the cell cycle in melanoma, colon, and pancreatic cancer tissues [33]. Dysregulation of CDK1 induced not only accelerated tumor growth but also sustained or spontaneous proliferation of cancer cells and even metastasis. As revealed, CDK1 was regarded as an oncogenic factor to be regulated by Vir Like M6A Methyltransferase Associated (VIRMA) in an N6-methyladenosine-independent manner in BC [34]. We demonstrated that CDK1 was upgraded in human BC tissues both in the transcriptional and translation levels, and high expression of CDK1 in breast tissues heralded poor prognosis of patients with BC. It is worth noting that overexpression of CDK1 increased tumorigenic potential and the capacity for tumor initiation [33]. Pharmacological inhibition of CDK1 decreased the phosphorylation level of CDK1, inhibited cell proliferation and invasion, and arrested the cell cycle at the G1 or G2/M phase in human cholangiocarcinoma cell lines [35]. Consistently, our *in vitro* functional experiment exhibited that knockdown of CDK1 suppressed the capacity of migration and invasion in MCF-7 and MDA-MB-231 cell line, indicative of an oncogenic role of CDK1 in the progression of BC.

CDC20 (cell division cycle 20 homologue) is a regulatory molecule performing critical activities in several parts of the cell cycle, human tumorigenesis and cancer progression [36]. As reported by Zhang et al., the patients with high expression CDC20 exhibited an association with more aggressive clinicopathological characters and worse prognosis in prostate cancer [37]. Our findings confirmed that high expression of CDC in patients had a low survival rate via bioinformatics analysis, and the expression of CDC20 was significantly increased in BC, and knockdown of CDC20 repressed the migration and invasion ability in MCF-7 and MDA-MB-231 cells via experimental validation. This conclusion is identical to a previous study that overexpression of CDC20 enhanced the metastatic capacity of MCF-7 cells, while inhibition of CDC20 by triterpenoid, a novel mushroom-derived CDC20 inhibitor, suppressed cellular metastatic capacity [22]. Taken together, aberrant expression of CDC20 was correlated with malignant progression and poor prognosis in BC.

AURKA, a subtype of serine/threonine kinases, plays an important role in the regulation of cell cycle and division [38]. AURKA is primarily localized at the poles of the mitotic spindle during mitosis, in which it serves to regulate the functionality of the centrosome and is requisite for the progression of mitosis [39]. AURKA was aberrantly expressed in many cancer cells, especially in gastric cancer [40]. Lately, a study suggested that AURKA acting as an oncogene increased RNase III DROSHA mRNA stability to transactivate STC1 expression through enhancement of N^6^-methyladenosine modification in BC stem-like cells [41]. In addition, in BC cells and human tissues, AURKA was markedly expressed via interaction with p-mTOR and p-ERK1/2, thereby promoting cell proliferation and migration [42]. Consistent with the results from former studies, we found that the expression of AURKA was increased both in microarray analysis and experimental validation, and was positively associated with a short overall survival rate, indicating that high expression of AURKA during BC played a detrimental role. Based on these previous studies and our results, AURKA could be a crucial factor in the progression of BC by regulating signaling pathways and might be a prospective biomarker and indicator of prognosis.

MCM4 belongs to the minichromosome maintenance (MCM) protein complex, which is composed of six well-conserved proteins (MCM2-7) operating together to initiate DNA replication and DNA depolymerization in response to their replicative helicase activity [43]. A study demonstrated that MCM4 was always expressed at a high level in BC of high histological grades, such as HER2-positive, and triple-negative subtypes of BC [44]. In addition, the low expression of MCM4 was regarded as an independent factor that correlates with an increased probability of relapse-free survival [44]. Similarly, Kwok et al. found that through analysis of 1441 patients with BC, increased expression of MCM4 was positively correlated with shorter survival [45]. Here, we found that the expression of MCM4 was dramatically upgraded in BC via microarray analysis and experimental validation, and a high level of MCM4 reduced the lifespan of BC patients to some extent. Together, MCM4 is a significative biomarker, and could be potentially a predictor of the development and prognosis in BC patients.

Several publications ever utilized GSE45827 and GSE124646 datasets to seek BC biomarkers. Studies have proved that high expression of AURKA, CDK1 and CDC20 is associated with poor overall survival in BC patients via analysis of the GSE45827 dataset [46,47]. However, they did not go further with their samples to verify the expression of these genes. In this study, we performed a comprehensive bioinformatics analysis and uncovered four hub genes (CDK1, CDC20, AURKA, and MCM4) that may be involved in BC carcinogenesis and progression. Moreover, we validated these four genes with samples from our BC patients and the results were consistent with the microarray analysis. Thus, our study not only verifies that these genes are associated with BC, but also provides more reliable and accurate results based on bioinformatics analysis and experimental validation.

## 5. Conclusion

Bioinformatical analysis delivers a convenient but efficient method to check out hypotheses of carcinogenic alterations, which helps investigators to transform basic studies into clinical applications. The present study demonstrated that increased CDK1, CDC20, AURKA, and MCM4 expression may be reliable and predictive biomarkers for poor prognosis in patients with BC. In addition, high expression of CDK1 and CDC20 are more susceptible to BC metastasis. Further studies should be focused on seeking precise mechanisms between these four hub genes and BC. All in all, these findings yield novel perspectives into the current understanding of four hub genes in BC.

## Supplementary Material

Supplemental Material

## Data Availability

The datasets used and/or analyzed during the current study are available from the corresponding author on reasonable request.

## Abbreviations

BC: Breast cancer; BP: biological process; CC: cellular components; DAVID: Annotation Visualization and Integrated Discovery; DEGs: Differentially expressed genes; GEO: Gene Expression Omnibus; GO: Gene Ontology; KEGG: Kyoto Encyclopedia of Gene and Genome; MCODE: Molecular Complex Detection; MF: molecular function; PPI: protein–protein interaction; STRING: Search Tool for the Retrieval of Interacting Genes.
