# Supplementary material for: Identification of novel biomarkers in breast cancer via integrated bioinformatics analysis and experimental validation
Source: Bioengineered. 2021 Dec 13;12(2):12431–46. doi: 10.1080/21655979.2021.2005747 (PMC8810011; doi:10.1080/21655979.2021.2005747)
Supplement: Supplemental Material [file KBIE_A_2005747_SM0574.docx]

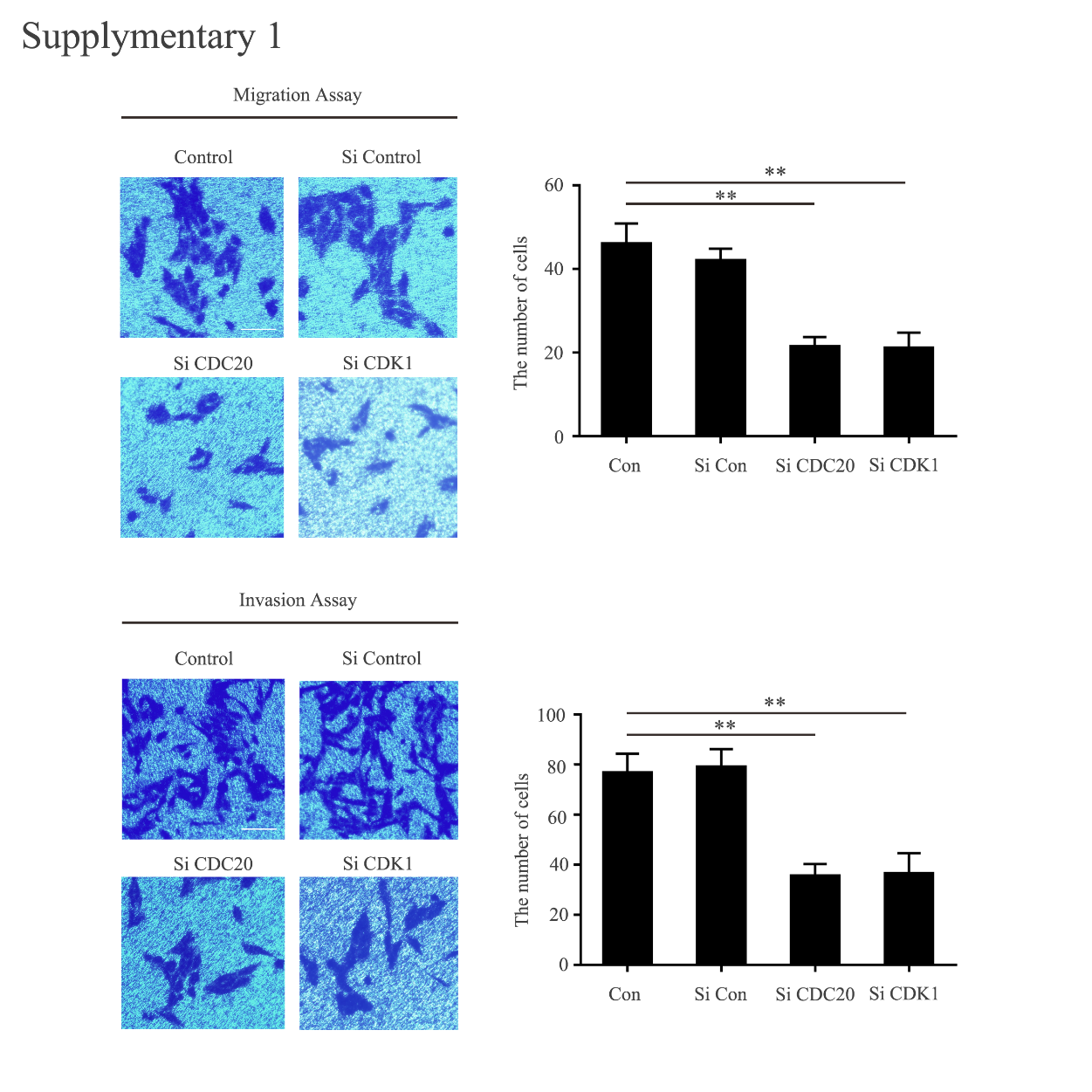


**Supplementary 1** **legend:** MDA-MB-231 cells were transfected with siRNA against human CDK1 (siCDK1) and CDC20 (siCDC20) or scrambled control siRNA, then incubated for 48 hours. Transwell and invasion assay were used for observing migration and invasion abilities of these cells. The images displayed the migrated and invaded cells into the lower chamber. (Scale bar=10 μm). Quantified by counting the number of migrated and invaded cells in five randomly fields. **P* < 0.05, ***P* < 0.005, ****P* < 0.001, compared with the control group.
